# Post‐Covid‐19‐vaccination adverse events and healthcare utilization among individuals with or without previous SARS‐CoV‐2 infection

**DOI:** 10.1111/joim.13453

**Published:** 2022-02-01

**Authors:** Francisco Tsz Tsun Lai, Lei Huang, Kuan Peng, Xue Li, Celine Sze Ling Chui, Eric Yuk Fai Wan, Carlos King Ho Wong, Esther Wai Yin Chan, Ivan Fan Ngai Hung, Ian Chi Kei Wong

**Affiliations:** ^1^ Centre for Safe Medication Practice and Research, Department of Pharmacology and Pharmacy, Li Ka Shing Faculty of Medicine The University of Hong Kong Hong Kong SAR China; ^2^ Laboratory of Data Discovery for Health (D24H), Hong Kong Science Park Hong Kong Science and Technology Park Hong Kong SAR China; ^3^ Department of Medicine, Li Ka Shing Faculty of Medicine The University of Hong Kong Hong Kong SAR China; ^4^ School of Nursing, Li Ka Shing Faculty of Medicine The University of Hong Kong Hong Kong SAR China; ^5^ School of Public Health, Li Ka Shing Faculty of Medicine The University of Hong Kong Hong Kong SAR China; ^6^ Department of Family Medicine and Primary Care, Li Ka Shing Faculty of Medicine The University of Hong Kong Hong Kong SAR China; ^7^ Research Department of Practice and Policy, School of Pharmacy University College London London United Kingdom

**Keywords:** Covid‐19, SARS‐CoV‐2 infection, vaccine safety

## Abstract

**Background:**

Post‐marketing pharmacovigilance data are scant on the safety of Covid‐19 vaccines among people with previous SARS‐CoV‐2 infection compared with ordinary vaccine recipients. We compared the post‐vaccination adverse events of special interests (AESI), accident and emergency room (A&E) visit, and hospitalization between these two groups.

**Methods:**

We conducted a retrospective cohort study using a territory‐wide public healthcare database with population‐based vaccination records in Hong Kong.

**Results:**

In total, 3922 vaccine recipients with previous SARS‑CoV‑2 infection and 1,137,583 vaccine recipients without previous SARS‑CoV‑2 infection were included. No significant association was observed between previous SARS‑CoV‑2 infection and AESI or hospitalization. Previous SARS‑CoV‑2 infection was significantly associated with a lower risk of A&E visit (CoronaVac: hazard ratios [HR] = 0.56, 95% confidence intervals [CI]: 0.32–0.99; Comirnaty: HR = 0.62, 95% CI: 0.47–0.82).

**Conclusion:**

No safety signal of Covid‐19 vaccination was detected from the comparison between vaccine recipients with previous SARS‐CoV‐2 infection and those without infection.

## Introduction

As of October 3, 2021, a total of 234 million people have been diagnosed with Covid‐19 worldwide [1]. Patients recovering from Covid‐19 typically develop antibodies and a certain degree of immunity against SARS‐CoV‐2, the causative agent of the disease, although evidence suggests that such protection may attenuate with time and reinfection is highly possible [2]. Recent research, nevertheless, has shown that certain vaccines are highly effective in boosting the immunity against the virus among Covid‐19 survivors [3].

Partly due to widespread concerns about vaccine safety, there is increasingly observed vaccine hesitancy in various world societies [4]. In particular, survey studies have shown that individuals with a previous SARS‐CoV‐2 infection show stronger hesitancy toward Covid‐19 vaccine uptake than those without a previous infection [5, 6]. A recent round of Gallup survey conducted in July 2021 showed that 18% of the respondents among the American population who did not intend to receive the vaccine believed that enough protection has already been acquired from a previous infection [7]. The same survey also suggested that the unclear safety profile of vaccines was an equally important reason (18%) for the nonuptake [7].

Data on the safety of vaccines among those with a previous SARS‐CoV‐2 infection as compared with other vaccine recipients are scant [8]. This current study aimed to use a territory‐wide public healthcare database linked with population‐based vaccination records in Hong Kong to detect any differences in the risk of adverse events of special interest (AESI), accident and emergency room (A&E) visit, and hospitalization between those with a previous infection and those without following vaccination. Currently, Comirnaty (Pfizer‐BioNTech) and CoronaVac Covid‐19 vaccines are approved for emergency use in Hong Kong.

## Methods

### Data source

Territory‐wide de‐identified electronic medical records between January 1, 2018, and July 31, 2021, were obtained from the Hospital Authority (HA), the statutory body managing all public hospital services. Population‐based vaccination records between February 23, 2021 (the launch date of the vaccination programme) and July 31, 2021 were obtained from the Department of Health (DH), the government health agency in charge of the Covid‐19 vaccination programme. Data linkage between the two data sources was performed by matching unique pseudo‐IDs to protect patient privacy. These data and linkage methods have been successfully used in identifying the association between Bell's palsy and CoronaVac vaccination previously [9]. This study was approved by the institutional review board of the University of Hong Kong/Hospital Authority Hong Kong West Cluster (UW 21–149 and UW 21–138) and the Department of Health Ethics Committee (LM 21/2021).

### Study design

This was a retrospective cohort study. As patients with a previous SARS‑CoV‑2 infection were recommended to take only one dose of the Covid‐19 vaccine in Hong Kong, the index date of the SARS‑CoV‑2 infection group was the date of taking the first dose of either CoronaVac or Comirnaty. For other vaccine recipients, the index date was the date of receiving the second dose of either CoronaVac or Comirnaty. The observation period of this study was 28 days. The primary outcome of this study was time to any AESI from the index date. A total of 30 conditions were considered as AESI, adapted from the World Health Organization's Global Advisory Committee on Vaccine Safety (Table S1). The secondary outcomes of this study were time to A&E visit, and time to hospitalization through A&E. For each outcome, the observation ended 28 days after the index date, upon death, or the end date of data availability (July 31, 2021), whichever was the earliest.

### Cohort selection

The study cohort consisted of two groups, the SARS‑CoV‑2 infection group, and the other vaccine recipient group. All patients with a positive result on the SARS‑CoV‑2 polymerase chain reaction (PCR) test who received one dose of either CoronaVac or Comirnaty after SARS‑CoV‑2 infection were included in the SARS‑CoV‑2 infection group. All patients with a negative result on the SARS‑CoV‑2 PCR test or with no SARS‑CoV‑2 PCR test result who were fully vaccinated with either CoronaVac or Comirnaty were included in the other vaccine recipient group. Patients with AESI history before the index date were excluded from the study cohort.

### Statistical analysis

We stratified the cohort by vaccine type. Propensity score weighting was implemented by the R package “WeightIt” to assign weights and generate balanced cohorts considering the potential confounding effects of age, sex, and history of chronic diseases. We calculated the Charlson Comorbidity Index to represent the history of chronic diseases, which takes the severity of diseases into consideration. The standardized mean differences (SMD) between the SARS‑CoV‑2 infection group and the other vaccine recipient group were examined with the SMD being smaller than 0.1 indicating balance between the groups. Cox proportional hazard models were used to examine the association between previous SARS‑CoV‑2 infection and study outcomes in the weighted cohorts, with patients without an infection history as the referent group.

## Results

**Fig. 1:**
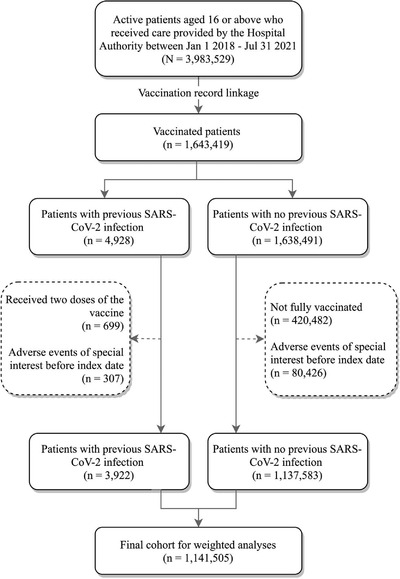
Cohort selection procedures.

After applying the eligibility criteria, the final cohort included 3922 vaccine recipients with previous SARS‑CoV‑2 infection (CoronaVac: 943; Comirnaty: 2979), and 1,137,583 vaccine recipients with no previous SARS‑CoV‑2 infection (CoronaVac: 511,802; Comirnaty: 625,781). Figure 1 shows the cohort selection procedures graphically. Table 1 shows the cohort characteristics by vaccine type. Vaccine recipients with previous SARS‑CoV‑2 infection tend to be younger than those with no previous SARS‑CoV‑2 infection. CoronaVac recipients, compared with Comirnaty recipients, were more likely to be male, at older age, and with more chronic conditions. After weighting, the maximum SMD for all baseline characteristics were smaller than 0.1.

**Table 1:** Baseline characteristics of patients with previous SARS‑CoV‑2 infection and other vaccine recipients by vaccine type

	CoronaVac		Comirnaty	
	Patient with previous SARS‑CoV‑2 infection	Patient without previous SARS‑CoV‑2 infection	Patient with previous SARS‑CoV‑2 infection	Patient without previous SARS‑CoV‑2 infection
*N*	943	511,802	979	625,781
Sex = Male (%)	411 (43.6)	244,550 (47.8)	1459 (49.0)	288,488 (46.1)
Age (mean (SD))	51.12 (13.00)	53.22 (13.67)	42.60 (14.77)	45.88 (14.95)
CCI (%)				
0	848 (89.9)	461,848 (90.2)	2768 (92.9)	583,535 (93.2)
1–2	91 (9.7)	47717 (9.3)	200 (6.7)	40,193 (6.4)
3–4	2 (0.2)	1126 (0.2)	7 (0.2)	913 (0.1)
≥5	2 (0.2)	1111 (0.2)	4 (0.1)	1140 (0.2)

Abbreviation: CCI, Charlson Comorbidity Index.

**Fig. 2:**
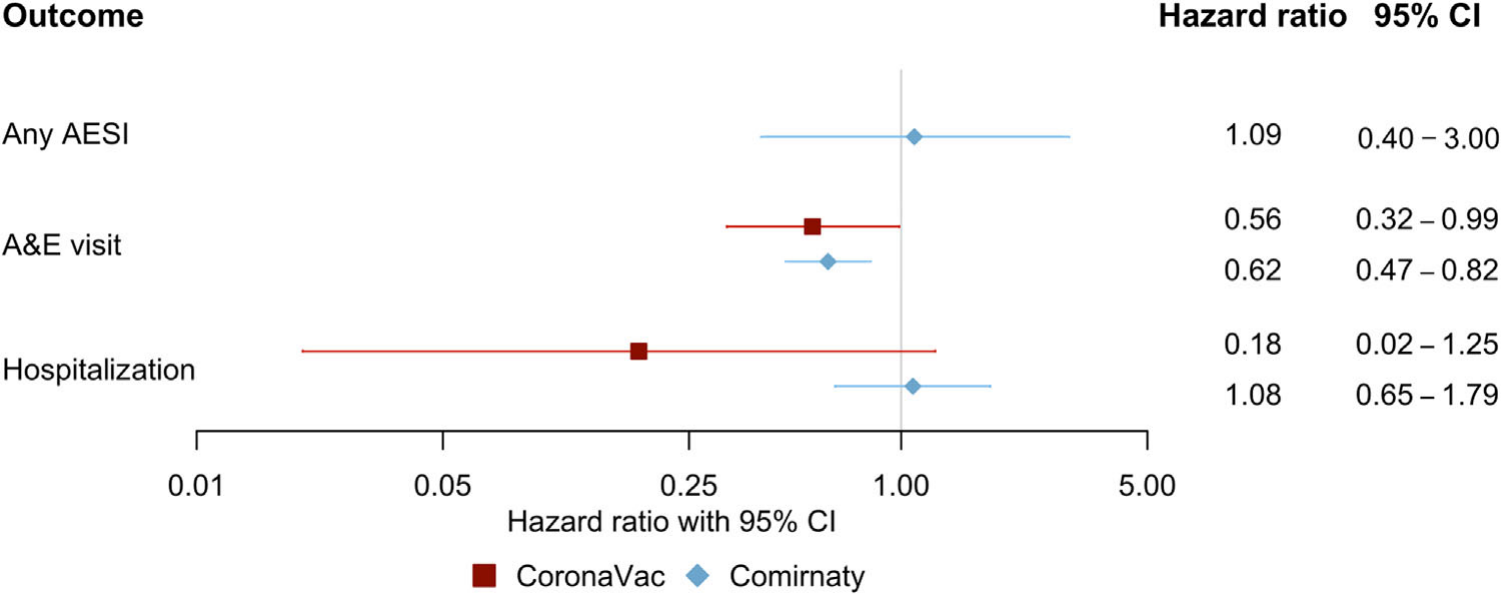
Forest plot showing hazard ratios with 95% confidence intervals (CI) for vaccine recipients with a previous SARS‑CoV‑2 infection compared with those without any previous infection receiving the second dose. AESI, adverse events of special interest; A&E, accident and emergency department.

Figure 2 shows the adjusted hazard ratios (HR) and corresponding 95% confidence intervals (CI) between vaccine recipients with and without previous SARS‑CoV‑2 infection on the outcomes. Within the observation period, 802 (0.16%) CoronaVac recipients and 851 Comirnaty (0.14%) recipients had AESI. Given that no CoronaVac recipient with previous SARS‑CoV‑2 infection developed AESI, further analysis was not conducted for this subgroup. For Comirnaty recipients, no significant association was observed between previous SARS‑CoV‑2 infection and AESI (HR = 1.09, 95% CI: 0.40–3.00). During follow‐up, 12,956 CoronaVac recipients (2.53%) and 17,822 Comirnaty recipients (2.83%) were admitted to the A&E department. For both vaccine types, previous SARS‑CoV‑2 infection was associated with a lower risk of A&E visit (CoronaVac: HR = 0.56, 95% CI: 0.32–0.99; Comirnaty: HR = 0.62, 95% CI: 0.47–0.82). There were 3129 CoronaVac (0.61%) and 3584 Comirnaty recipients (0.57%) being hospitalized through A&E. No significant association between previous SARS‑CoV‑2 infection and hospitalization through A&E visit was observed for either CoronaVac (HR = 0.18, 95% CI: 0.02–1.25) or Comirnaty recipients (HR = 1.08, 95% CI: 0.65–1.79).

## Discussion

We did not identify a higher risk of AESI, A&E visit, and hospitalization following Covid‐19 vaccination among individuals with a previous SARS‐CoV‐2 infection compared with other vaccine recipients after their second dose. In fact, a lower risk of A&E visit was observed among those with an infection history for both vaccine types.

This lower risk was likely due to better follow‐up care services provided to those with a previous infection. The prevailing policy in Hong Kong has always been institutionalizing all people infected with SARS‐CoV‐2, with varying extent of medical attention and intervention provided depending on the severity of the disease. As the infection rate in Hong Kong has largely been kept under control, with the daily number of new cases never exceeding 200 out of almost 7.5 million people, healthcare professionals are often able to closely follow up with all patients recovering from Covid‐19. Thus, many minor symptoms could have been addressed in follow‐up consultations without seeking help from A&E. Our findings should be reassuring to people having recovered from Covid‐19 in that the safety profile of the vaccines is likely also applicable to people with an infection history. The results are also in line with the existing literature with no specific risks identified for people with previous infection receiving the vaccine, except research on self‐reported reactogenicity showing an increased risk of milder adverse reactions that require no medical intervention [10].

Limitations to the study should be noted despite the absence of a safety signal. First, AESI may be handled in settings beyond the local public healthcare system, such as the private sector or overseas. Nevertheless, in terms of number of hospital admissions that are warranted for most of the included AESI, the HA constitutes approximately 80% of the market share in Hong Kong. Second, residual confounding is probable because the variety of covariates considered in the analysis may not be sufficiently wide. Third, we used SARS‐CoV‐2 PCR test to indicate SARS‐CoV‐2 infection, but a positive result on the PCR test is not equivalent to an infection. Last, as the population of Hong Kong is predominantly Chinese, replication of the analyses in other world populations is warranted to test for generalizability of the results.

In conclusion, no safety signal of Covid‐19 vaccination was detected from the comparison between vaccine recipients with previous SARS‐CoV‐2 infection and those without infection.

## Conflict of interest

F.T.T.L. has been supported by the RGC Postdoctoral Fellowship under the Hong Kong Research Grants Council and has received research grants from the Food and Health Bureau of the Government of the Hong Kong Special Administrative Region, outside the submitted work. C.S.L.C. has received grants from the Food and Health Bureau of the Hong Kong Government, Hong Kong Research Grant Council, Hong Kong Innovation and Technology Commission, Pfizer, IQVIA, and Amgen, and personal fees from PrimeVigilance outside the submitted work. E.Y.F.W. has received research grants from the Food and Health Bureau of the Government of the Hong Kong Special Administrative Region, and the Hong Kong Research Grants Council, outside the submitted work. XL has received research grants from the Food and Health Bureau of the Government of the Hong Kong Special Administrative Region; research and educational grants from Janssen and Pfizer; internal funding from the University of Hong Kong; and consultancy fees from Merck Sharp & Dohme, unrelated to this work. E.W.Y.C. reports honorarium from Hospital Authority; and grants from Research Grants Council (RGC, Hong Kong), Research Fund Secretariat of the Food and Health Bureau, National Natural Science Fund of China, Wellcome Trust, Bayer, Bristol‐Myers Squibb, Pfizer, Janssen, Amgen, Takeda, and Narcotics Division of the Security Bureau of the Hong Kong Special Administrative Region, outside the submitted work. I.C.K.W. reports research funding outside the submitted work from Amgen, Bristol‐Myers Squibb, Pfizer, Janssen, Bayer, GSK, Novartis, the Hong Kong Research Grants Council, the Food and Health Bureau of the Government of the Hong Kong Special Administrative Region, National Institute for Health Research in England, European Commission, and the National Health and Medical Research Council in Australia; he has also received speaker fees from Janssen and Medice in the previous 3 years and is an independent non‐executive director of Jacobson Medical in Hong Kong. All other authors declare no conflict of interest.

## Author Contributions

Francisco Tsz Tsun Lai: conceptualization, methodology, writing original draft, reviewing and editing. Lei Huang: methodology, formal analysis, writing original draft, reviewing and editing. Kuan Peng: formal analysis and validation. Xue Li: supervision, reviewing and editing. Celine Sze Ling Chui: supervision, reviewing and editing. Eric Yuk Fai Wan: supervision, reviewing and editing. Carlos King Ho Wong: supervision, reviewing and editing. Esther Wai Yin Chan: funding acquisition, supervision, reviewing and editing. Ivan Fan Ngai Hung: supervision, reviewing and editing. Ian Chi Kei Wong: funding acquisition, supervision, reviewing and editing.

## Supporting information


**Supplementary Table 1**: List of AESI and corresponding diagnosis codes.

## Data Availability

Data are not available as the data custodians have not given permission for sharing.
